# Noisy condition and three-point shot performance in skilled basketball players: the limited effect of self-talk

**DOI:** 10.3389/fspor.2023.1304911

**Published:** 2024-01-10

**Authors:** Liu Yang, Yu Tian, Yingchun Wang

**Affiliations:** ^1^ School of Psychology, Beijing Sport University, Beijing, China; ^2^ School of Psychology, Shanghai University of Sport, Shanghai, China; ^3^ Tsinghua University High School, Beijing, China

**Keywords:** self-talk, motor performance, cognitive distraction, noise, psychological skill

## Abstract

In modern basketball, the three-point shot plays an important tactical role. Basketball players often face the distraction from audience and opponents, necessitating psychological skill to maintain their performance. The study examined the effects of self-talk interventions on the three-point shot performance under quiet and noisy conditions. It involved 42 national second-level basketball players and used a 2 (Condition: quiet condition, noisy condition) × 3 (Intervention: control group, motivational self-talk, instructional self-talk) mixed design to investigate the performance of the static and dynamic three-point shots tasks. The results revealed that the static three-point shot score was significantly lower in noisy condition compared to quiet condition (*p* = 0.016), while the main effect of Intervention and the interaction effect of Condition × Intervention were not significant. *Post-hoc* analysis indicated that only the control group showed significantly lower scores in the noisy condition (*p* = 0.043). For the dynamic three-point shots performance, there were no significant main effects of Intervention or Condition, nor any significant interaction effect between Condition and Intervention. In conclusion, noise distraction negatively affects the static three-point shots task, and although self-talk interventions can mitigate such negative effects, their effectiveness is limited for dynamic three-point shots task with high physical demands.

## 1 Introduction

In over 130 years since basketball's inception, numerous rule and tactical changes have emerged. Prominent among these is the introduction of the three-point shot during the 1979–1980 National Basketball Association (NBA) season. Presently, the three-point shot holds significant tactical importance in basketball, with teams focusing extensively on it to enhance their probability of winning (1, 2). In the NBA, elite teams often prioritize outside offensive strategies, such as three-point shooting, to exploit their advanced skill sets and shooting accuracy. On the other hand, less dominant teams typically emphasize inside plays, focusing on scoring from closer to the basket through layups, dunks, and post moves. This approach often relies more on physicality and less on shooting prowess (2). The statistical data derived from the Basketball World Cup 2019 also demonstrated that the number of three-point shots in the final had a positive relationship with winning the game (1). In modern basketball, the traditional distinctions of on-court positions have become increasingly fluid. Players are now expected to possess a broader range of skills, with even conventional centers trying their hand at three-point shots (3). This emphasis that the three-point shot is increasingly recognized as a key tactical element, reflecting a trend that appears to align with evolving strategies and game dynamics, highlighting its role in enhancing team performance and success.

The tactical importance of the three-point shot in basketball is clear, but successfully executing it during a game involves overcoming several challenges, including external distractions (4). Factors like noise or light can potentially hinder motor performance, a common issue for basketball players who often face noise distractions from audiences or rival teams. On the field, noise levels can be considerably high; for example, in sports such as American football, audience peak noise levels can reach an astonishing 123–140 dB (5). Such intense noise levels not only impede athletes’ ability to communicate but also pose a rigorous test to their performance, as indicated by research findings. For instance, a study by Galanis and Hatzigeorgiadis revealed that, despite 6 weeks of shooting training, there was a significant reduction in shooting accuracy when athletes were subjected to noise distractions as compared to baseline levels where no such distraction was present (6). According to Attentional Control Theory (ACT), auditory noise disrupts attentional control processes, making it difficult to focus on the task at hand (7, 8). This disruption compromises athletes’ attentional control ability, hindering their concentration on movements or strategies during competitions, and ultimately results in poorer performance (8). The degree of disruption is further exacerbated by the unpredictability and variability of the noise. Constant noise might be easier to adapt to or ignore, whereas sudden or varying noises are more likely to capture attention and disrupt performance (9). Summarily, performance decrements under auditory distractions are attributed to the involuntary diversion of attention, the subsequent reallocation of attentional resources, and individuals’ metacognitive evaluations of their coping capabilities. Key psychological characteristics such as commitment, motivation, and determination are essential. These traits, as highlighted in studies on elite musicians and athletes, play a pivotal role in not just acquiring but also manifesting expertise in high-pressure environments (10). Consequently, this underscores the necessity for basketball players to develop robust psychological skills, alongside physical skill training, to counteract the detrimental effects of noise distractions.

One of the most frequently employed mental skills in sports is self-talk, which takes various forms (11). Self-talk can be categorized into motivational self-talk (e.g., “I can do it”) and instructional self-talk (e.g., “elbow, wrist, shoot”) based on its strategic characteristics (12). Theodorakis, Weinberg (12) suggest that instructional self-talk is more effective in fine motor control compared to motivational self-talk, whereas motivational self-talk is superior in strength and endurance sports. To gain a deeper insight into the impact of self-talk on basketball performance, we reviewed all research containing the terms “self-talk” and “basketball” and summarized 7 quantitative studies from 2001 to 2020 (Table 1). In studies examining the effects of self-talk on student participants, one research involving 60 students found that the self-talk word “relax” notably enhanced shooting task performance compared to the “fast” and control groups (13). In a parallel study of 72 students, participants using Instructional Self-Talk (IST) demonstrated superior accuracy in both passing and shooting tasks, while those adopting Motivational Self-Talk (MST) achieved faster speed passing than the control group (14). For basketball players, self-talk strategies aid in regulating emotional states and enhancing performance.

**Table 1:** Quantitative studies on self-talk in basketball.

Year	Author(s)	Participants	Interventions	Cue-words	Measures	Outcome
2001	Theodorakis et al.	60 students	Self-talk	“Relax” and “fast”	Shooting task (Shoot for 3 min from five specified positions)	Word “relax” improved their performance significantly as compared to the control group and “fast” group
2011	Boroujeni & Shahbazi	72 students	IST and MST	IST: move your fingers to target carefully; MST: I can move my fingers to target carefully	Pass tasks (accuracy and speed) and shooting task (shoot from various marked positions)	IST group had better performance than the other groups in accuracy pass and shot; MST group performed speed passing faster than control group
2017	Altfeld et al.	20 youth basketball players	Self-regulation skills (e.g., self-talk, self-relaxation, routines)	None	German volitional components questionnaire sport	Self-regulation skills help players regulate their emotional state
2018	Abdoli et al.	20 professional basketball players	IST and MST	IST: ring front, elbow, wrist; MST: I will be successful	Free throw task	IST increases the shooting accuracy and decreases movement coordination variability compare to baseline
2018	Galanis et al.	28 female basketball players	Combination of IST and MST	IST: focus, rim; MST: it is in, count it	Free throw task	Self-talk group performed better than participants of the control group
2019	Amado et al.	The sample consisted of 191 athletes and 30 (15.7%) were basketball players	None	None	Behavioral regulation in sport questionnaire	Enhancing more positive self-talk may promote a better performance
2020	Einarsson et al.	The sample consisted of 396 athletes and 89 (44.9%) were basketball players	None	None	Test of performance strategies questionnaire	Older athletes use psychological skills training (i.e., self-talk) more

IST, instructional self-talk; MST, motivational self-talk.

Consistent practice of self-talk influences motivation and dispositional state-orientation after failure (15). This finding is corroborated by a qualitative study. In competitive situations, self-talk assists athletes in regulating cognition and behavior, enhancing motivation, and managing affect (16, 17). In exploring the effectiveness of self-talk interventions in basketball, various studies have employed different methodologies. For instance, a study combined IST and MST, found this intervention to be effective in enhancing free throw performance, even amidst distractions (6). However, the study did not determine which form of self-talk intervention was more effective. Another study involved IST and MST interventions separately, where basketball players with IST demonstrated improved free throw accuracy and reduced movement coordination variability, a measure obtained using biomechanical analysis techniques, indicating more consistent and fluid movements (18). In contrast, MST showed no significant influence on these outcomes (18). Additionally, questionnaire-based studies have been conducted to explore the relationship between self-talk frequency and athletic performance. One study involving 191 athletes from team sports used questionnaires to assess the impact of self-talk on performance and anxiety control. It found that the satisfaction of basic psychological needs, particularly autonomy, was a strong predictor of positive self-talk during competition. This study relies on self-reported measures, while providing valuable insights, raises questions about the subjective interpretation of self-talk and its effects (19). Another study, focusing on the psychological skills of 396 Icelandic athletes, used the Test of Performance Strategies questionnaire (The Cronbach's alpha values were 0.69 for competition items and 0.75 for practice items). This study showed a substantial interest in Psychological Skills Training (PST), with notable differences in psychological skills among athletes based on their use of PST and gender. While the study provided a broad view of psychological skills usage, including self-talk, its cross-sectional nature and the self-assessment approach limit the longitudinal applicability and objectivity of the findings (20). While these studies collectively suggest a positive influence of self-talk, particularly instructional self-talk, on basketball performance, their reliance on self-reported data and varying experimental controls raise questions about reliability and validity. This underscores the need for more rigorous, methodologically diverse approaches to strengthen the evidence base in this field.

In line with this need for methodological diversity, research on self-talk in basketball motor performance, however, still predominantly focuses on the free throw task rather than three-point shots. From a physical perspective, shooting from the three-point area not only requires a faster release speed and maintenance of the proper flight angle and shot direction but also superior cardiopulmonary capacity, which has a more stringent demand than a free throw (21, 22). In modern basketball, emphasizing three-point shooting attempts is an essential part of game preparation, as successful three-point shots correlate positively with winning (1). As the game of basketball continues to evolve, keeping pace with the current trends and shifts in modern basketball will ensure that the study of self-talk remains relevant, applicable, and beneficial to both coaches and players. With the increasing physical and psychological demands of the game, understanding the role and impact of self-talk can be a vital component in optimizing performance and fostering a positive mental environment for athletes (6, 14, 16, 18).

Given the significance of three-point shots in basketball and the presence of disruptive noise in competitive settings, this study seeks to investigate the effects of specific conditions (i.e., noisy/quiet conditions) and self-talk strategies (i.e., IST/MST) on three-point shooting performance. Given that both kinematic skills and anaerobic capacities play roles in three-point shots, IST and MST might produce distinct outcomes in varying scenarios and task demands. Specifically, IST reduces movement coordination variability, whereas MST counteracts the negative effects of mental fatigue on endurance performance (18, 23, 24).

Drawing upon the literature reviewed and the identified research gaps, this study explored the effects of MST and IST compared to a control group, both in quiet and noisy environments, as well as potential interactions between these conditions and self-talk strategies. We employed two tasks (i.e., static and dynamic shooting tasks) on the basketball court to examine the effects of interventions under conditions of low and high physical fitness demands, respectively. These methods, to a certain degree, offer a more realistic representation of athletes’ in-game scenarios. Based on previous findings, our hypotheses are as follows: (1) participants exhibit lower scores under noisy condition in static and dynamic three-point shots tasks; (2) self-talk affects three-point shot performance and moderates the effects of noise distractions on it. Specifically, in the static three-point shots task, only the IST group improved shooting performance compared to the MST and control groups under noisy condition. In contrast, for the dynamic three-point shots task, the MST group showed improved performance relative to the IST and control groups in the noisy condition.

## 2 Method

### 2.1 Participants

The sample size was computed by G*Power 3.1, by selecting “ANOVA: Repeated measure, within-between interaction” as statistical test, effect size *f* = 0.25, *α err prob* = 0.05, *power (1-β err prob)* = 0.80, number of groups = 3, number of measurements = 2, and keeping the rest of the parameters default (25). Based on these parameters, a total of 42 subjects were calculated to be necessary for the study. Forty-two participants (all males, age = 20.93 ± 2.50, Mean ± SD) who came from Beijing Sport University participated in this study, they were randomly assigned to 3 groups (i.e., IST, MST and control groups). All participants were national second-level basketball players (i.e., they participated in the National A or B-Level Leagues, National Youth League, or placed in the top ranks of provincial or national championships) and had normal or corrected-to-normal visual acuity. They have played basketball for 7.55 ± 4.13 (Mean ± SD) years and trained for 10.93 ± 6.22 (Mean ± SD) hours per week. The study was approved by the Sports Science Experiment Ethics Committee of Beijing Sport University (ethical identification number: 2022107H). All participants gave a written informed consent that they took part in the study voluntarily. As a reward for their participation, they were given a gift after the experiment.

### 2.2 Experimental design

Two (Condition: quiet condition, noisy condition) × three (Intervention: IST, MST, control group) mixed experimental design was used. The Condition is within-subjects variable and the Intervention is between-subjects variable. The dependent variables were static and dynamic three-point shots score.

### 2.3 Experimental materials

#### 2.3.1 Self-talk intervention

MST group used “I can” or “I can do it” as their self-talk cue (11). Considering the longer distance to the basket for the three-point shot compared to a free throw, it's essential to effectively transfer lower body strength and ensure the precision of the shot (26, 27). The IST group employed “tighten core, elbow wrist” as their self-talk cue. Meanwhile, the control group did not receive any intervention throughout the entire experiment.

#### 2.3.2 Noisy condition

The noise distraction material comprises basketball commentary, the sound of basketball dribbling, whistles from the audience, and recorded voices (6). The duration of the noise material is 4 min and 58 s. It is designed to be played continuously while the participant is performing the task. If the task duration exceeds 4 min and 58 s, the noise material should be replayed from the beginning and continue until the task is completed. The basketball commentary, excerpted from a particular game segment, spans a duration of 4 min and 58 s without any interruption. It maintains a moderate volume level and is presented in Chinese. The sound of basketball dribbling occurs at a frequency of once every second, with the volume set to the minimum level. The whistles from the audience manifest intermittently, with intervals ranging from 30 to 50 s, and are presented at the highest volume level. The recorded voices are comprised of “red, green, blue, up, down, right, miss the shot” in Chinese. They play uninterruptedly for a duration of 4 min and 58 s. The volume level is set to be lower than the basketball commentary but higher than the sound of basketball dribbling. The audio material was played on AirPods Pro in transparency mode. The playback intensity was monitored using the accessibility function provided by Apple's iOS operating system, with the actual playback intensity ranging from 85 to 95 dB (28).

### 2.4 Measures

#### 2.4.1 Free throw task

The participant stood at the free throw line and took a free throw after receiving a pass from the experimenter. A scoring system from Hardy and Parfitt (29) was used. A score of 0 is given for a complete miss (i.e., an air ball), 1 for a shot that hits the backboard and bounces out, 2 for one that hits the rim and bounces out, 3 for a shot that hits the backboard and goes in, 4 for one that hits the rim and goes in, and 5 for a “clean” basket. Participants made a total of 20 attempts, with the maximum achievable score being 100 points.

#### 2.4.2 Static three-point shots task

The participant, positioned at the top of the arc (6.75 m away and perpendicular to the basket), attempted a shot after receiving a pass from the experimenter. The scoring system mirrored that of the free throw task. Each set consisted of 10 shots, offering a potential maximum of 50 points.

#### 2.4.3 Dynamic three-point shots task

**Figure 1:**
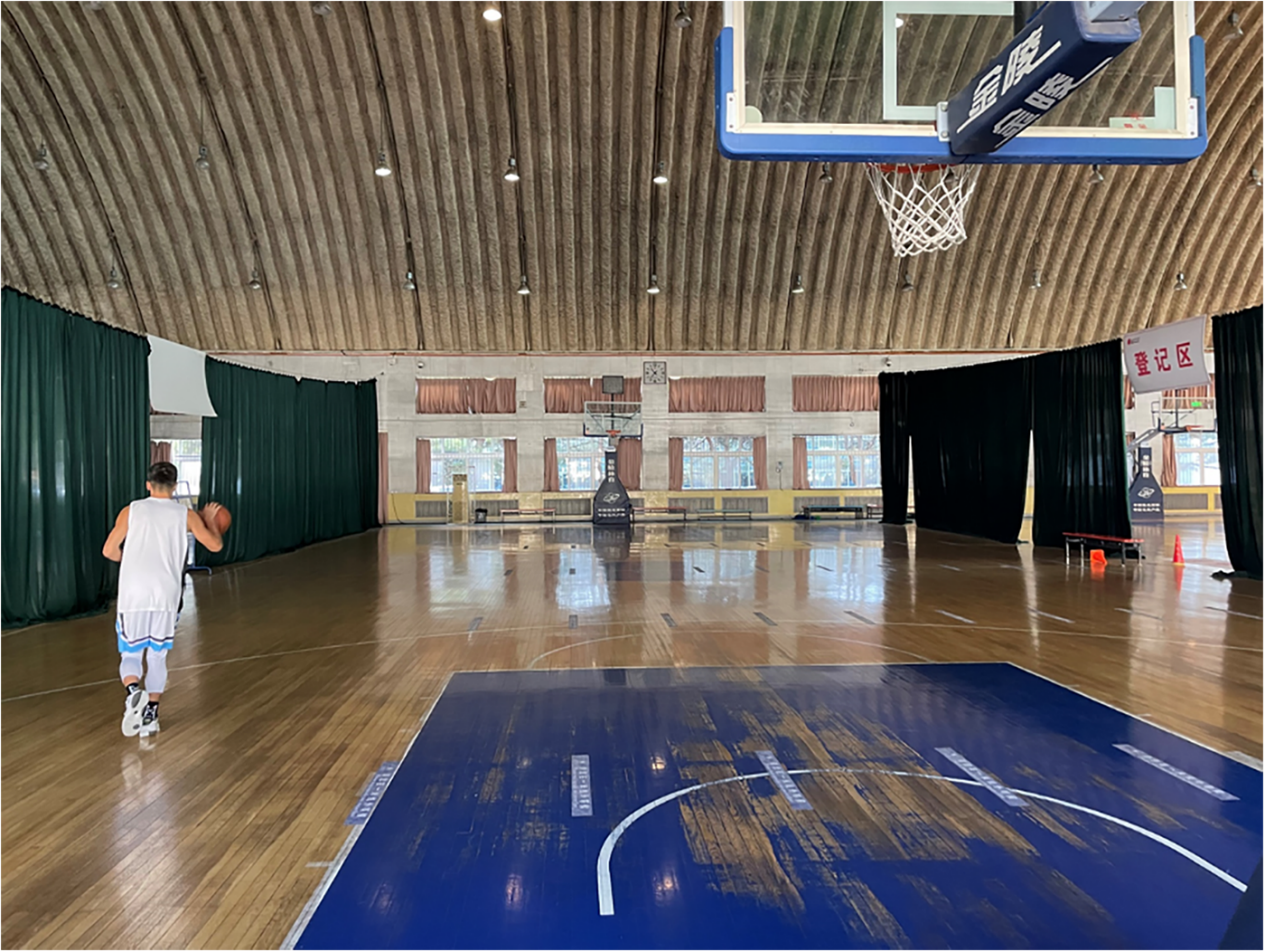
Dynamic three-point shots task. The participant in the image has just finished grabbing the rebound from the initial shot and is now dribbling out beyond the three-point line to attempt a second shot. Consent for the use of this image in this article has been obtained from the participant.

The participant takes a shot from any position outside the three-point line, then they ran to catch the rebound and dribbled out of the three-point line to attempt another three-point shot, repeating this process for 2 min. The participants were informed to make as many successful shooting attempts as possible. The scoring system was the same as the free throw task, with no maximum score cap (Figure 1).

#### 2.4.4 Manipulation check

A self-designed self-talk manipulation check questionnaire was used to assess the use of self-talk by the participants during task execution, including two items: (1) I said things like “I can” or “I can do it” to myself when shooting; (2) I said things like “tighten core, elbow wrist” to myself when shooting. A 5-point Likert scale was used, with 1 indicating “never” and 5 indicating “always.”

### 2.5 Procedure

The participants began with a 10 min warm-up, which included dynamic stretching and free shooting practice. They then undertook a free throw task with 20 attempts. Following this, a self-talk intervention was introduced. We initially introduced the concept and advantages of self-talk (i.e., “Self-talk is a psychological skill that involves silently or loudly speaking specific cue words to oneself during the course of a sporting activity. Existing research indicates that self-talk can facilitate performance in both gross motor skills and fine motor skills”). Following the introduction, we conducted an interactive session where participants could ask questions and express any concerns, allowing us to confirm their comprehension of the self-talk. Once understanding was confirmed, we moved to the practical application phase. Participants were instructed to practice three-point shooting and were required to employ self-talk cues before each shot. This exercise aimed to integrate the self-talk skill into their shooting action, providing a real-time application of the self-talk. The effectiveness of the intervention was monitored by observing the participants’ adherence to using self-talk cues during the practice session. Next, participants engaged in a static three-point shooting task divided into 4 sets: 2 in quiet conditions and 2 in noisy conditions, arranged in an ABBA counterbalanced design. The same design was followed for the dynamic three-point shots task. After completing the tasks, participants filled out a questionnaire, which included demographic information and a manipulation check. They were also asked questions regarding their use of self-talk during the tasks.

### 2.6 Data analysis

The data was analyzed using SPSS 26.0. Initially, an ANOVA was conducted to examine the manipulation check and demographic information. The reliability of the shooting performance measures was assessed using the Intraclass Correlation Coefficient (ICC) for both static and dynamic tasks. Lastly, a 2 × 3 repeated measures analysis of variance (RMANOVA) was carried out, with Intervention (IST, MST, control group) as the between-subjects factor and Condition (quiet, noisy condition) as the within-subjects factor.

## 3 Results

### 3.1 Manipulation check and basketball-related information

A one-way ANOVA was conducted on the manipulation check, revealing significant differences between different interventions for both item 1 [self-talk cue about “I can” or “I can do it” during shooting; *F* (2, 39) = 64.11, *p* < 0.01] and item 2 [self-talk cue about “tighten core, elbow, wrist” self-talk during shooting; *F* (2, 39) = 50.30, *p *< 0.01]. Utilizing the Bonferroni-adjusted method for post-hoc pairwise comparisons. For the item 1, a significant difference was observed between control group and MST [*p* < .001, 95% CI (−3.38, −2.19)], and between IST and MST [*p* < .001, 95% CI (−3.74, −2.26)]. The comparison between control group and IST was not significant [*p* = 1.00, 95% CI (−0.53, 0.95)]. For the item 2, there was a significant difference between control group and IST [*p* < .001, 95% CI (−2.95, −1.48)], and between MST and IST [*p* < .001, 95% CI (−3.52, −2.05)]. The difference between control group and MST was not significant [*p *= .176, 95% CI (−0.16, 1.31)]. Descriptive statistics are provided in Table 2. The results indicated that self-talk manipulation was successful.

**Table 2:** Description statistics of basketball-related information and manipulation check.

Intervention	NOP	PBY M (SD)	THPW M (SD)	Item 1 M (SD)	Item 2 M (SD)	FTS M (SD)
CG	14	7.50 (4.40)	9.86 (3.23)	1.57 (0.85)	1.86 (0.95)	70.57 (8.17)
MST	14	6.07 (3.58)	12.21 (7.43)	4.36 (0.75)	1.29 (0.61)	70.07 (6.47)
IST	14	9.07 (4.10)	10.71 (7.29)	1.36 (0.75)	4.07 (0.73)	70.07 (10.04)
Sum	42	7.55 (4.13)	10.93 (6.22)	2.43 (1.58)	2.40 (1.43)	70.57 (8.16)

NOP, number of participants; PBY, playing basketball years; THPW, training hours per week; Item 1, I said things like “I can” or “I can do it” to myself when shooting; Item 2, I said things like “tighten core, elbow wrist” to myself when shooting; FTS, free throw score; CG, control group; MST, motivational self-talk; IST, instructional self-talk.

One-way ANOVA was also conducted to examine the differences among the self-talk interventions in terms of basketball-related information. The results revealed that there was no significant difference in playing basketball years among the self-talk interventions [*F* (2, 39) = 1.93, *p* = 0.159]. Furthermore, there was no significant difference in training hours per week among the self-talk interventions [*F* (2, 39) = 0.50, *p* = 0.609], nor was there any significant difference in free throw score [*F* (2, 39) = 0.05, *p* = 0.951]. Descriptive statistics are also provided in Table 2. The results indicate that there were no statistical differences among the groups in terms of basketball-related information.

### 3.2 Intraclass correlation coefficients for static and dynamic three-point shots tasks

In the analysis of shooting task performance, the Intraclass Correlation Coefficients (ICC) for the static and dynamic three-point shots task were calculated. The ICC for single measures in the static three-point shots task was 0.26 [95% CI (0.11, 0.43)]. For average measures, a moderate reliability was observed with an ICC of 0.58 [95% CI (0.33, 0.75)]. The F-tests for single and average measures were significant [*F* (41, 123) = 2.38, *p* < .001], implying reliable measurements beyond chance.

The dynamic shooting task demonstrated greater reliability, with a single measures ICC of 0.68 [95% CI (0.55, 079)], and average measures ICC of 0.89 [95% CI (0.83, 094)]. The corresponding F-tests were significant [*F* (41, 123) = 9.31, *p* < .001], underscoring the consistency of performance in the dynamic three-point shots task.

### 3.3 The effects of condition and intervention on three-point shots performance

**Figure 2:**
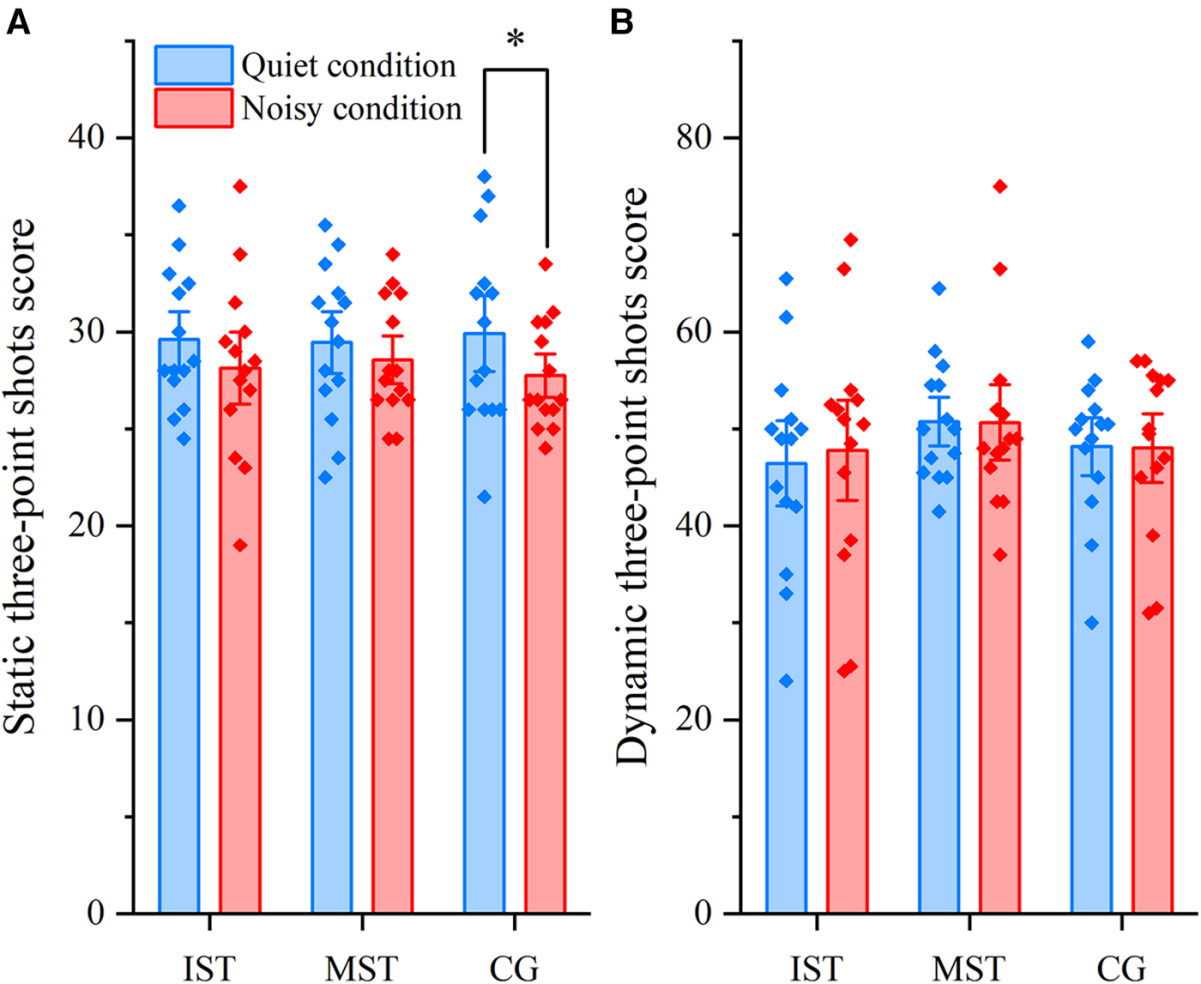
Comparison of conditions and self-talk interventions on basketball performance. (**A**) Results of static three-point shots score (*p < 0.05). (**B**) Results of dynamic three-point shots score. The blue column represents quiet condition, the red column represents noisy condition. Each point represents the mean score for each participant. CG, control group; MSTG, motivational self-talk group; ISTG, instructional self-talk group. The error bar represents standard error.

Results from the mixed-design ANOVA revealed a significant main effect of Condition on static three-point shots [*F* (1, 39) = 6.31, *p* = 0.016, partial *η*^2^ = 0.139, *power* = 0.688]. However, there was no significant main effect for Intervention [*F* (2, 39) = 0.01, *p* = 0.989, partial *η*^2^ = 0.001, *power* = 0.052], nor was there a significant interaction effect between Condition and Intervention [*F* (2, 39) = 0.38, *p* = 0.685, partial *η*^2^ = 0.019, *power* = 0.107] for static three-point shots. Although the interaction was not statistically significant, we pursued a simple effects analysis to delve deeper into potential influences. The Bonferroni adjustment indicates a significant result with a *p*-value less than 0.05; notably, it revealed significant differences in the control group's performance between the noisy and quiet conditions [*p* = .043, 95% CI (0.07, 4.29)]. However, neither the MST [*p* = .397, 95% CI (−1.22, 3.00)] nor the IST [*p* = .168, 95% CI (−0.65, 3.57)] showed significant differences in scores under the noisy condition (Figure 2A). For the scores on dynamic three-point shots, neither the main effects of Condition [*F* (1, 39) = 0.12, *p* = 0.728, partial *η*^2^ = 0.003, *power* = 0.064], nor Intervention [*F* (2, 39) = 0.60, *p* = 0.555, partial *η*^2^ = 0.030, *power* = 0.142] reached statistical significance. Additionally, there was no significant interaction effect between Condition and Intervention [*F* (2, 39) = 0.21, *p* = 0.815, partial *η*^2^ = 0.010, *power* = 0.080; see Figure 2B].

## 4 Discussion

### 4.1 Effects of noise distraction and self-talk on performance

In high-intensity basketball games, athletes are not only under immense pressure but are also subject to constant interference from opponents and spectators. This places a significant demand on their shooting ability in order to maintain a high level of performance. This study examined the effects of noise distraction and self-talk on three-point shot performance based on a simulated competition scenario. The study designed to enhance the ecological validity and closely replicated the physical demands and pace of an actual game. Static and dynamic three-point shot tasks were conducted, with the former characterized by a lower level of physical demand, while the latter required participants to engage in continuous play involving shooting, rebounding, and dribbling. The results partially supported Hypothesis 1, indicating that auditory distractions impair static three-point shot performance, yet had no significant effect on dynamic three-point shot performance under higher physical demands. However, we failed to support Hypothesis 2 (i.e., self-talk moderates the negative effects of auditory distractions on performance) and did not replicate the findings of previous research, which indicated that both MST and IST positively affect motor performance (6, 13, 18, 30, 31).

Specifically, the results showed a significant main effect of conditions on the scores of static three-point shots, but not on the scores of dynamic three-point shots. In the noisy condition, the performance on static three-point shots was significantly lower than that in the quiet condition. This finding is consistent with previous studies indicating that noise distraction affects basketball shooting performance (6). One possible reason is that noise distraction diminishes attention ability, leading to a reduction in performance. A meta-analysis of 242 studies found a significant negative effect of noise distraction on cognitive performance, with an effect size of d = −0.31 [95% CI (−0.42 to −0.25)], suggesting a general impairment of cognitive functions such as attention ability due to noise (28). Additionally, the highest effect size for high loudness, short duration, *d* = −0.68 [95%CI ( −0.96 to −0.40)], suggesting that short, loud noises have the most negative effect on attentional control (28). In our study, this phenomenon is reflected in the context of static three-point shots performance, where noise distraction could similarly impair the cognitive processes involved in shooting, such as attention. This aligns with the meta-analysis, as participants faced an environment with noise designed to replicate the auditory distractions in a competitive basketball game, thereby extending the implications of noise distraction from cognitive tasks to motor performance in sports. Klostermann (32) found that attentional control positively predicted basketball free throw accuracy, with prolonged Quiet Eye duration as shooting accuracy improved. The enhancement of visual attention through Quiet Eye training has been shown to increase the precision of basketball three-point shots while also mitigating the effects of attentional disruptions that arise when performing under high-pressure conditions (33). Additional evidence from the study on verbal distraction indicated that such distraction can interfere with free throw execution and lead to less accurate movements by drawing attentional resources away from the task (34). Therefore, in the static three-point shot task, noise distraction may weaken attentional ability, causing participants to allocate more attention to extraneous stimuli and impeding the execution of the intended motor action.

However, the lack of a significant difference in the dynamic three-point shot performance between quiet and noisy conditions was in conflict with the results of previous studies (6, 35, 36). It should be noted that the dynamic three-point shot task has not been previously investigated, and the physical demands of this task differ from those of traditional basketball performance tasks or other sports performance tasks. Therefore, the absence of a significant difference in athlete performance between quiet and noisy conditions is not unexpected. Moreover, there is evidence that noise distraction does not affect the performance of expert athletes (37, 38). Both Hassmén and Koivula (38) and Herrebrøden, Sand Sæbø (37) have reported no significant effect of noise on the athletic performance of professional golf players. First possible reason is motivation, athletes generally have a higher level of motivation than the general population, which is one of their special characteristics (39, 40). The athletes may have been highly motivated to perform well regardless of the noise level, and this motivation may have overridden the negative effects of noise. As Herrebrøden, Sand Sæbø (37) suggested, experts can cope with auditory distractions due to their advanced skills might be able to perform more confidently when noise distractions are present.

Second possible reason is arousal, the dynamic three-point shot task required higher physical demands, which may have caused relatively higher arousal level among the athletes compare to static three-point shot task. Moderate levels of arousal led to optimal performance (41). The athletes may have been able to maintain their attention on the task at hand regardless of the noise distraction during the dynamic three-point shot task.

Unexpectedly, the self-talk interventions did not significantly affect the static and dynamic three-point shots performance, and we failed to conduct interactions between Condition and Intervention. However, these results do not imply that self-talk interventions are completely ineffective. Specifically, in the static three-point shot performance, the control group scored lower in the noisy condition, whereas this pattern did not occur for the motivational and instructional self-talk groups.

### 4.2 Applied implications

These findings indicate that self-talk can help counter the effects of external distractions and maintain performance during low physical demands (6). However, the results from Galanis, Hatzigeorgiadis (6) are inconsistent with the dynamic three-point shots performance. This suggests that there is still controversy over the effects of self-talk interventions on basketball shooting performance. Abdoli, Hardy (18) suggested that skill level and task complexity should be considered more closely. More complex skills, the better developed understanding of the task requirements held by elite performers, enable them to more use instructional self-talk effectively than novices (18). In the present study, firstly, the participants were athletes who had proficient mastery of three-point shooting skills and reached a level of automation in motor execution. Secondly, the tasks were three-point shooting tasks, which differed from previous free throw tasks, as it required a higher level of accuracy and consistency in shooting technique. The long-term intervention was more successful than the short-term intervention, including state self-confidence, self-efficacy, self-optimization, and coach-rated performance (42). In the present study, however, athletes only used self-talk before shooting, and there was no long-term self-talk intervention conducted. Additionally, for dynamic shooting task, intensity and time pressure are high, motor execution relies on automation; for static shooting task, although motor execution also relies on automation, there is sufficient preparation time in each trail and thus is more susceptible to interference from irrelevant internal or external stimuli (43). In conclusion, for the high intensity three-point shooting performance of basketball players, an acute self-talk intervention may not have an immediate effect on improving their performance.

### 4.3 Strengths, limitations, and future research

The present study explored the relationship between noisy condition, self-talk and three-point performance in-depth. Firstly, the study utilized a randomized controlled design and met the sample size calculated by G*power, which ensures the statistical power is reliable. Secondly, the study focused on skilled basketball players, which enhances the applicability of the findings to similar populations. Thirdly, the use of dynamic three-point shots tasks in this study, which have not been used by previous researchers, provides a more realistic competition scenario and higher ecological validity.

However, there are several limitations in the present study. According to attentional control theory, anxious individuals will increase motivation as a means of compensation to keep performance (7). Lacking of measure of motivation, state anxiety and other confounding variables in the present study. It is recommended to include subjective or objective indicators to control confounding variables in future studies. In addition, the present study solely measured the performance of the three-point shot and lacked more sophisticated indicators, such as motor coordination variability or quiet eye. Therefore, it is recommended that future studies incorporate more refined indicators to investigate the effects of noisy condition and self-talk on sports performance. While we believe our simulation provides an approximation of a competitive scenario, we acknowledge certain limitations inherent to a laboratory setting. One such limitation is the absence of defenders, who are integral to competitions. Defenders influence shooting performance through several behavioral variables, such as execution times and movement variability (44). Future research could aim to include defender simulations to further enhance the ecological validity of the task. Finally, all participants in the study were male basketball players, and there was a lack of female participants. Therefore, caution is needed when extrapolating the study results to females. It is recommended that future studies include female athletes as participants, or compare male and female athletes to investigate potential gender differences.

## 5 Conclusion

In conclusion, the present study investigated the effects of noise distraction and self-talk on basketball players’ three-point shots performance. Results indicated that the static three-point shots performance was significantly affected by the noisy condition, while no significant differences were observed in the dynamic three-point shot performance. The study also found that self-talk was an effective strategy for counteracting the negative effects of noise distraction in the static three-point shots task. Consequently, coaches or athletes may benefit from incorporating self-talk as a training strategy for increasing shooting performance, especially in low physical demand training situations. However, the study results indicated no significant effect of self-talk on the performance of dynamic three-point shots task, which suggests the need for the combination of three-point shot skills training and long-term self-talk interventions to improve performance in high physical demands situations.

## Data Availability

The original contributions presented in the study are included in the article/Supplementary Material, further inquiries can be directed to the corresponding author.
